# Virus-specific T cells and neutralizing antibodies are independent correlates of long-term protective immunity against hepatitis C virus

**DOI:** 10.1128/jvi.00849-26

**Published:** 2026-09-10

**Authors:** Elsa Gomez-Escobar, Asiyah Siddique, Omar Khedr, Nathalie Bédard, Alizée Fieffé-Bédard, Nourtan F. Abdeltawab, Nicol Flores, Mohamed S. Abdel-Hakeem, Mohamed Eisa, Geng Zang, John Lok Man Law, Juthika Kundu, Michael Houghton, Julie Bruneau, Naglaa H. Shoukry

**Affiliations:** 1 Centre de Recherche du Centre Hospitalier de l’Université de Montréal (CRCHUM) 177460, Montréal, Quebec, Canada; 2 Département de microbiologie et immunologie, Université de Montréal 5622, Montréal, Quebec, Canada; 3 Department of Microbiology and Immunology, Faculty of Pharmacy, Cairo University, Giza, Egypt; 4 School of Pharmacy, Newgiza University, Giza, Egypt; 5 Department of Medical Microbiology and Immunology, Li Ka Shing Applied Virology Institute, University of Alberta 3158, Edmonton, Alberta, Canada; 6 Département de médecine familiale, Université de Montréal 5622, Montréal, Quebec, Canada; 7 Département de médecine, Université de Montréal 5622, Montréal, Quebec, Canada; Wake Forest University School of Medicine, Winston-Salem, North Carolina, USA

**Keywords:** chronic infection, people who inject drugs, T cells, neutralizing antibodies, hepatitis C

## Abstract

Virus-specific T cells and broadly neutralizing antibodies (NAbs) are associated with spontaneous clearance of acute primary hepatitis C virus (HCV) infection and reinfection. Following spontaneous clearance, HCV-specific memory T cells are long-lived, but NAbs are less durable. How these two arms contribute to long-term protective immunity upon re-exposure is unknown. Herein, we compared the magnitude and breadth of memory HCV-specific T cells and NAbs in a cohort of HCV-spontaneously resolved people who inject drugs with high-risk exposure to the virus but who remained free of observed reinfections (FOR, *n* = 34) or who got reinfected (*n* = 22). Among FOR, 73.5% (*n* = 25) exhibited high frequencies of gamma interferon (IFN-γ)-producing T cells, while 23.5% (*n* = 8) showed high neutralization breadth (>25% neutralization of HCV pseudoparticles [HCVpp]) and/or potency (geometric mean neutralization >50% against seven HCVpp). Notably, some subjects had detectable NAbs several years after clearance. Multivariate random forest analysis demonstrated that the frequency of IFN-γ-producing T cells and the NAb response against only one difficult-to-neutralize HCVpp (1b58) were significant predictors of protection against reinfection (*P* < 0.05). Longitudinal analysis of the immune response in subjects with multiple episodes of infection showed that strong and broad HCV-specific T cell responses were associated with spontaneously cleared episodes. In contrast, NAbs did not protect from chronicity upon re-exposure in the absence of memory T cells, or if they were unable to recognize the infecting virus due to imprinting by strains of the previous episodes. In conclusion, T cell responses and NAbs targeting specific isolates are independent predictors of long-term protective immunity against HCV.

**IMPORTANCE:**

Hepatitis C virus (HCV) is a blood-borne virus that disproportionately affects people who inject drugs. Most HCV infections become persistent, leading to liver fibrosis and cancer. Although effective antiviral therapies are available, around 50 million people remain persistently infected with HCV and can transmit the virus. Currently, there is no available vaccine to control the spread of HCV. However, around one in four people infected can naturally clear the virus, becoming partially protected upon re-exposure, suggesting it is possible to develop a vaccine that induces similar protection. In this study, we investigated the contribution of T cells and neutralizing antibodies to protection against observed reinfection in a cohort of people who inject drugs and have naturally cleared HCV, yet remain at high-risk exposure. Our findings suggest that strong T cell responses and neutralizing antibodies targeting hard-to-neutralize isolates could predict protective immunity upon reinfection.

## INTRODUCTION

Hepatitis C virus (HCV) infection resolves spontaneously in ~25% of cases, while the rest develop persistent infection and progressive liver disease (1). Despite the availability of effective direct-acting antivirals (DAA), the asymptomatic nature of the infection, combined with limited screening and access to treatment in vulnerable populations, leaves a significant proportion of HCV-chronically infected individuals undiagnosed and untreated, representing a potential source of new infections and/or reinfections (2). In the absence of any prophylactic vaccine, the incidence of new HCV infections is on the rise in association with the opioid epidemic (3, 4). HCV reinfection following spontaneous clearance ranges from 1.8 to 46.8 cases per 100 person-years (5). Furthermore, sustained virological response following DAA treatment does not protect against reinfection, especially among people who inject drugs (PWID) (6).

PWID who have spontaneously cleared a prior HCV infection are 12 times more likely to clear the infection again than HCV-naive PWID with similar risk exposures, with ~80% of secondary infections getting resolved, compared to only 25% following a primary infection (7–9). These data suggest long-lived protective immunity against HCV. Virus-specific CD4 and CD8 T cell responses are essential for spontaneous clearance of acute infection (10) and are expanded and correlate with resolution of reinfection (9, 11). Moreover, neutralizing antibodies (NAbs) correlate with the spontaneous clearance of primary HCV infection (12–15) and with reduced magnitude and duration of viremia upon reinfection (9). In chimpanzee and humanized mouse models of HCV infection, prevention or attenuation of acute infection by NAbs has been clearly demonstrated (16–19). Finally, coordinated virus-specific T cell expansion and NAbs are associated with spontaneous clearance of primary HCV infection and reinfection, suggesting that both arms of immunity are essential for long-term protection (20, 21).

To date, there is still no vaccine against HCV. The only prophylactic vaccine that went into a phase I/II clinical trial is a T-cell-based vaccine targeting HCV non-structural proteins using a recombinant chimpanzee adenovirus 3 vector priming followed by a recombinant modified vaccinia Ankara boost (22). This vaccine did not protect against chronic infection in high-risk PWID, yet it was associated with lower viral loads in the vaccinated group, suggesting a partial protective effect of T cells. Multiple new-generation vaccines are in development targeting T cells and/or NAbs in different combinations. Yet, the contribution of each of these two arms to long-term protective immunity is not clear. This study aimed to examine the T cell and NAb response after spontaneous clearance and whether they protect against reinfection upon real-life exposure in PWID, a setting that mimics exposure post-vaccination. Our data suggest that a high frequency of HCV-specific T cells and NAbs are independent contributors for long-term protection against HCV reinfection.

## MATERIALS AND METHODS

### Study subjects

**Fig 1:**
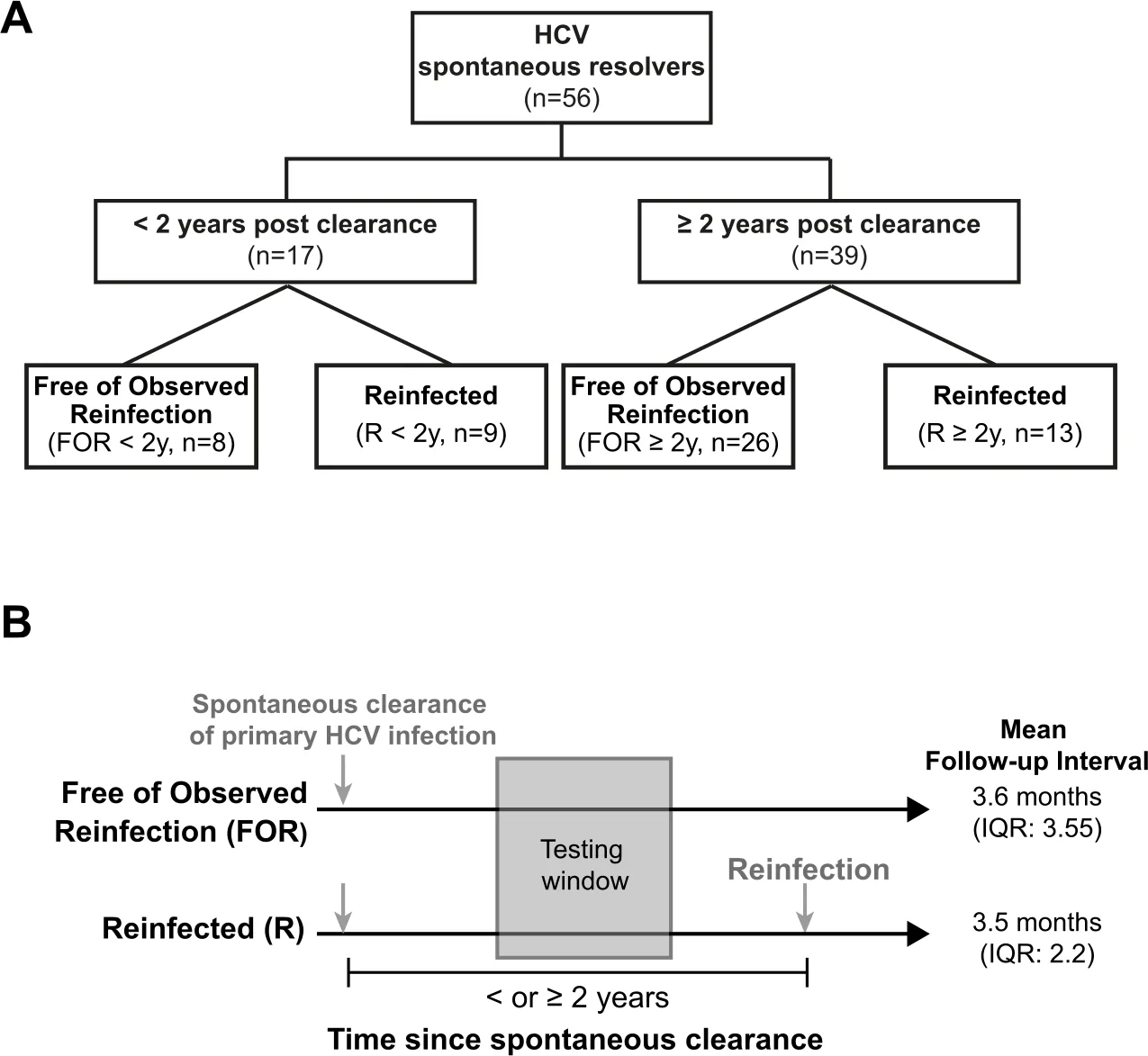
Flowchart detailing the classification of participants in the study. (**A**) HCV spontaneous resolvers were followed up and classified according to the time of follow-up after spontaneous clearance (<2 or ≥2 years). Then, we further categorized them into free of observed reinfection (FOR) or reinfected (R). (**B**) Schematic representation of the testing window for each group of participants. The mean follow-up interval is indicated for each group along with the interquartile range (IQR).

Study design is presented in Fig. 1A. PWID who had previously resolved one HCV infection episode spontaneously were recruited via the Montreal Hepatitis C Cohort as previously described (23). To be eligible, participants had to report drug injection in the past 6 months, given that injection drug use is the primary risk factor for HCV exposure. Participants also had to report at least one high-risk behavior: (i) sharing of material used for the consumption of psychoactive substances, (ii) recent incarceration, or (iii) injection of prescribed opioids and cocaine in the past month. For the present study, participants had to be spontaneous resolvers of HCV primary infection. Of the 56 spontaneous resolvers, 38 subjects were recruited already as spontaneous resolvers, 12 subjects as naive, and 6 subjects as acute HCV. *IFNL4* (previously called *IL28B*) genotype (rs12979860 polymorphism) was determined as previously described (24). Subjects’ clinical history was reviewed to exclude those with prior HCV treatment. HCV resolvers were identified as HCV-RNA negative using the COBAS Ampliprep/COBAS Amplicor HCV Test, version 2.0 (Roche Molecular Systems) (sensitivity, 50 IU/mL) and anti-HCV antibody positive by AxSym HCV Assay (Abbott GmbH & Co.). History of previous infections was compiled to define the date of clearance of primary infection. For subjects where the exact dates of primary infection and clearance were unknown, we estimated the time of their first infection using an epidemiological paradigm that predicts the date of HCV infection based on time since the beginning of injection drug use. According to Hagan et al. (25), it takes approximately 5 years to become infected with HCV since the initiation of injection drug activity. To examine long-term immunity and account for the possibility of viral rebound with the first 2 years of infection (8), subjects were further stratified according to time (<2 or ≥2 years) since primary infection resolution. All subjects were followed to detect cases of reinfection by retesting for HCV RNA every 3 months for a period up to 24 months. Participants exposed to HCV but free of observed reinfection (FOR) were defined as never having an HCV RNA-positive test after a resolved primary infection. Primary infection was defined by an HCV Ab-positive or HCV RNA-positive test, while HCV reinfection was defined by an HCV RNA-positive test after two negative tests at least 60 days apart. The median between the last negative and first positive HCV RNA test was used to determine the estimated date of reinfection (EDI). Spontaneous resolution of reinfection was declared if HCV RNA was no longer detectable upon two consecutive negative HCV RNA tests >30 days apart (6 months post-EDI). Chronic HCV reinfection was defined as a positive result from an HCV RNA test >6 months after EDI (Fig. 1). In subjects with multiple episodes of reinfection, we analyzed the following time points during each episode: early acute (<3 months post-EDI), late acute (3–7 months post-EDI), follow-up (7–12 months post-EDI), and late follow-up (>1-year post-EDI).

### IFN-γ enzyme-linked immunospot assay

Gamma interferon (IFN-γ) enzyme-linked immunospot (ELISpot) assay was performed against 11 pools of overlapping peptides representing the genotype 1a (H77) or the genotype 3a (K3a) polyprotein as previously described (26). Peptides were obtained from the Biodefense and Emerging Infections Research Resources Repository (BEI Resources). Briefly, all assays were performed in duplicate directly *ex vivo* on 2 × 10^5^ cryopreserved peripheral blood mononuclear cells (PBMCs). Specific spot-forming cells (SFCs) were calculated as the mean number of spots in test wells minus the number of spots in negative control wells and normalized to SFC/10^6^ PBMCs. The positive threshold was set as 160 SFC/10^6^ PBMCs based on the average of the sum of the response to the 11 peptide pools plus two standard deviations from 10 HCV-naive subjects. Breadth was defined as the number of proteins of the HCV polyprotein (Core, E1E2, p7/NS2, NS3, NS4A/B, NS5A, and NS5B) targeted. If the genotype of the primary infection was unknown, genotype 1a (the most predominant genotype in North America [27]) peptides were used.

### Generation of HCV pseudoparticles

HCV pseudoparticles (HCVpp) were produced by lipofectamine-mediated transfection as previously described (21, 28). CD81-KO HEK293T cells were transfected with pNL4-3.Luc.R-E-plasmid containing the env-defective HIV proviral genome and a luciferase reporter, pAdVantage, and HCV E1E2 plasmids (UKNP1.11.6, 1a154, UKNP4.2.2, 1a72, 1b58, UKNP3.1.2, or UKNP1.18.1) (kindly provided by Dr. Justin Bailey, Johns Hopkins School of Medicine) (29). Mock HCVpp were generated without the HCV E1E2 plasmid and used as controls.

### HCVpp neutralization assays

Neutralization assays were performed as previously described (21, 28). Huh-7 cells (1.5 × 10^4^ per well) were plated on sterile 96-well white plates (Thermo Fisher Scientific) and incubated overnight at 37°C. Fixed dilutions (1:25) of heat-inactivated plasma were pre-incubated at 37°C for 1 h with an equal volume of HCVpp (Tier 1: UKNP1.11.6; Tier 2: 1a154; Tier 3: UKNP4.2.2, 1a72, and 1b58; Tier 4: UKNP3.1.2 and UKNP1.18.1; or mock) (final plasma dilution 1:50). HCV-negative and HCV-positive (chronically infected donor) plasma were used as negative and positive controls, respectively. Plasma-HCVpp mixture was added to Huh-7 cells in duplicate and incubated for 5–6 h at 37°C. Then, the mixture was replaced with phenol-red-free DMEM (Wisent) and incubated for 72 h at 37°C. HCVpp entry was defined as the luciferase activity in Huh-7 cell lysates expressed in relative light units (RLUs) measured using a Varioskan Lux Multimode Microplate Reader (Thermo Fisher Scientific). Percent neutralization was calculated as [1 – (RLU_time point_ – RLU_mock_)/(RLU_negative_– RLU_mock_)]. Neutralization breadth was defined as the number of HCVpp neutralized >25%, cut-off previously established (29–32), and neutralization potency was calculated as the geometric mean of percent neutralization from the seven HCVpp used for a given time point.

### HCV E2 and NS3 ELISA

ELISA was performed as described previously (20) with 0.5 μg/mL of recombinant in-house generated H77-E2 and J6-E2 (a kind gift from Dr. Arash Grakoui, Emory University, Atlanta, GA, USA) (33) or NS3 protein [Raybiotech, genotype 1a (H77), amino acids 1192–1459]. Plasma samples from the subjects were diluted starting at 1:250 with fourfold dilutions up to 1:16,000. All samples were tested in duplicate. The difference in optical densities (OD450−570) was determined by subtracting the mean absorbance at 570 nm (background) from the mean absorbance at 450 nm. Absorbance measurements were performed on a Synergy 4 Microplate Reader (Biotek Instruments). The area under the curve (AUC) was calculated in Prism version 10.4.1 (GraphPad). The positive threshold was set at 84.3 (H77-NS3), 304.9 (H77-E2), and 1,133.3 (J6-E2), based on the average AUC plus two standard deviations measured in samples from 10 HCV-naive subjects.

### Tetramers

The MHC class I monomers for the HLA-B27-restricted HCV NS5B peptide aa 2841–2849 (ARMILMTHF) (B27/NS5B-2841) were synthesized at the NIH Tetramer Core Facility, Emory University, Atlanta, GA, USA. Tetramers were prepared by adding 10 μL of phycoerythrin-labeled ExtrAvidin (Sigma) five times, with 10-min incubation at room temperature after each addition. Tetramer staining was performed as previously described (34).

### Activation-induced marker assay

The activation-induced marker (AIM) assay was performed as previously described (21, 35). Briefly, cryopreserved PBMCs were thawed with RPMI 5 (RPMI medium supplemented with 5% human serum [Wisent] and 1% penicillin/streptomycin [Wisent]). PBMCs (2 × 10^6^ cells) were incubated for 3 h at 37°C in polypropylene push cap tubes (Thermo Fisher Scientific). A CD40 blocking antibody (Miltenyi) was then added to prevent CD40L-CD40 interaction and the subsequent downregulation of CD40L. Cells were stimulated for 18 h at 37°C, with either 1 µg/mL of HCV NS5B overlapping peptide pools of genotype 1a or 3a, or with HCV NS5B_2841–2849_ (aa 2841–2849 [ARMILMTHF]) minimal peptide (10 µg/mL). For the dose-response experiment, we used 20, 10, 5, and 2.5 µg/mL of minimal peptide, peptide containing the NS5B epitope ARMILMTHF (genotype 1a), and peptide containing the variant epitope VRMVMMTHF (genotype 3a). Staphylococcal enterotoxin B (Toxin Technology Inc.)-stimulated and unstimulated cells served as the positive and negative controls, respectively. Following stimulation, cells were stained for 30 min at 4°C with viability dye (LIVE/DEAD Fixable Aqua Dead Cell Stain Kit [Thermo Fisher Scientific]) and surface markers (Table S1) and fixed using 1% formaldehyde. Multiparameter flow cytometry data were acquired at the CRCHUM flow cytometry core using a LSRFortessa (BD Biosciences) and analyzed using FlowJo software (version 10.8.1 for Mac; BD Biosciences). AIM^+^ cells were defined as cells co-expressing CD69^+^4-1BB^+^ after stimulation.

### Statistical analysis

Statistical differences between groups were calculated using the Kruskal-Wallis test followed by Dunn’s multiple comparisons test or using the two-tailed Mann-Whitney test. The relationships between variables were examined using Spearman’s rank correlations in Prism version 10.4.1 (GraphPad). Multivariate random forest classification models were adjusted in R in order to predict which group (FOR <2 years, FOR ≥2 years, R <2 years, or R ≥2 years) a particular subject pertained to. For the adjustment and optimization of the models’ hyperparameters, the libraries ranger and caret were used in conjunction. The aforementioned optimization was done using threefold repeated (*n* = 5) cross-validation to overcome any challenges that may arise relating to the sample size. For assessment and visualization of variable importance, the packages randomForest, rfPermute, and ggplot2 were used. All statistical analyses were conducted using the R programming language, version 4.2.2. For a detailed description, please refer to the supplemental material.

## RESULTS

### Cohort characteristics and study time points

The aim of this study was to delineate correlates of protective immunity in a cohort of PWID who have spontaneously resolved one HCV infection but were still at risk of reinfection due to unsafe injection drug use. HCV-resolved subjects were identified and followed for incidence of reinfection as described in Materials and Methods. Participants’ demographics and clinical characteristics are listed in Table 1. Subjects were stratified into two groups depending on their risk exposure (through active unsafe drug injection) to HCV after spontaneous clearance: short-term (<2 years, *n* = 17) and long-term (≥2 years, *n* = 39) (Fig. 1A). To evaluate the protective immunity, we further classified them into free of observed reinfection or reinfected (R): FOR <2 years (*n* = 8), R <2 years (*n* = 9), FOR ≥2 years (*n* = 26), and R ≥2 years (*n* = 13). Spontaneous resolution of primary HCV infection is documented as a negative HCV RNA test result (HCV Ab+). For each subject, we analyzed one time point during this post-spontaneous resolution phase and stratified participants depending on where this point is situated relative to the spontaneous resolution: FOR <2 years (7–19 months), FOR ≥2 years (2–24 years), and R <2 years or ≥2 years (closest date before incidence of reinfection) (Fig. 1B). FOR and R participants reported an equivalent number of injections during the month prior to the selected time point, suggesting a comparable exposure and risk of reinfection (Table 1). Groups with long-term risk exposure to HCV (FOR ≥2 years and R ≥2 years) had longer intervals for follow-up visits compared to the standard 3-month follow-up period, suggesting participants in these two groups possibly missed visits and may have had undetected infections (Fig. S1A). However, since these subjects are distributed among both the FOR and R sub-groups, it is unlikely that this longer follow-up interval influenced the outcome of re-exposure.

**TABLE 1:** Clinical characteristics of spontaneously resolved subjects^*a*^

Characteristic	Free of reinfection, *n* = 34	Reinfected, *n* = 22
Sex (M/F)	27/7	19/3
Median age at time point post-clearance (range)	41 years (21–50)	34 years (20–56)
HCV genotype primary infection (1/3/NA)	8/1/25	5/2/15
IFNλ4 rs12979860 (CC/CT/TT/NA)	16/9/2/7	10/7/3/2
Number of injections during past month (<30/≥30)	15/19	8/14
Mean follow-up interval (range; IQR)	3.6 months (1–8.2; 3.55)	3.5 months (1–8.6; 2.2)
Outcome of reinfection (SR/CI)	Not applicable	14/8

^*a*^ NA, not available; SR, spontaneous resolver; CI, chronic infection; IQR, Interquartile range.

### PWID with long-term HCV exposure and free of observed reinfection have superior HCV neutralization activity

**Fig 2:**
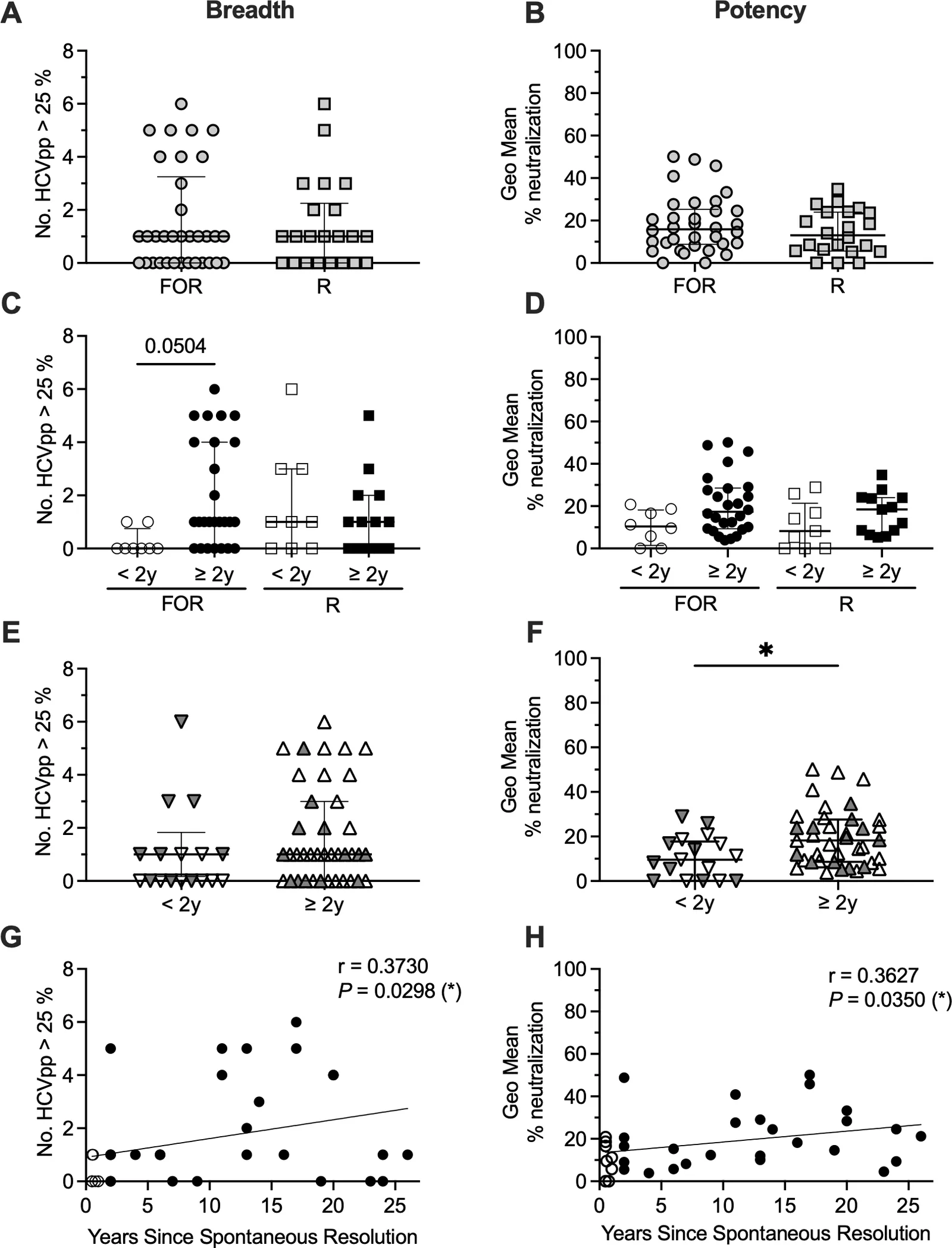
PWID with long-term HCV exposure and free of observed reinfection have superior HCV neutralization activity. (**A and B**) Neutralization breadth (A, number of HCVpp neutralized >25%) and neutralization potency (B, geometric mean of percent neutralization against seven HCVpp) of free of observed reinfection (*n* = 34) and reinfected (*n* = 22) subjects after spontaneous clearance of primary HCV infection. Two-tailed Mann-Whitney test. (**C and D**) Neutralization breadth (**C**) and neutralization potency (**D**) of FOR and R subjects stratified by time since spontaneous clearance: FOR <2 years (*n* = 8), FOR ≥2 years (*n* = 26), R <2 years (*n* = 9), and R ≥2 years (*n* = 13). Kruskal-Wallis test with subsequent Dunn’s multiple comparisons. (**E and F**) Comparison of neutralization breadth (**E**) and neutralization potency (**F**) between all FOR (open symbols) and R (closed symbols) subjects exposed <2 years or ≥2 years. Two-tailed Mann-Whitney test, *P < 0.05. (**G and H**) Scatter plots for Spearman’s rank correlation between the years post-spontaneous clearance and the neutralization breadth (**G**) or neutralization potency (**H**) for FOR subjects (*n* = 34): FOR <2 years (open circles) and FOR ≥2 years (closed circles). The *r*- and *P*-values are shown. *P < 0.05.

First, we measured the plasma neutralization activity for all participants against seven HCVpp harboring HCV envelope glycoproteins with different neutralization sensitivities from a standardized panel (tiers 1–4, where 4 is the most difficult to neutralize) (29). As a positive control, we used plasma from a chronically infected subject who neutralized HCVpp in a tier-dependent manner (Fig. S2). FOR and R subjects did not differ significantly in neutralization breadth (number of neutralized HCVpp >25%) and potency (geometric mean of percent neutralization) (Fig. 2A and B), even when stratified into subgroups (<2 years or ≥2 years) (Fig. 2C and D). However, there was a trend of higher neutralization breadth and potency in FOR subjects exposed ≥2 years compared to their <2-year counterparts (*P* = 0.0504; Fig. 2C). This trend was also observed in the neutralization of HCVpp tiers 2, 3, and 4 (Fig. S3). Because of that, we compared neutralization data by exposure time regardless of the reinfection status. We observed that only neutralization potency was significantly higher in subjects exposed for ≥2 years compared to those exposed for <2 years (*P* = 0.0185) (Fig. 2E and F). Finally, neutralization breadth and potency in FOR subjects correlated with time after spontaneous clearance (Fig. 2G and H). These results suggest that plasma neutralizing activity can be detected several years after spontaneous clearance in some long-term exposed PWID and may contribute to long-term protection from reinfection.

### HCV-specific T cells are long-lived, broad, and present in high frequency in exposed and free of observed reinfection PWID

**Fig 3:**
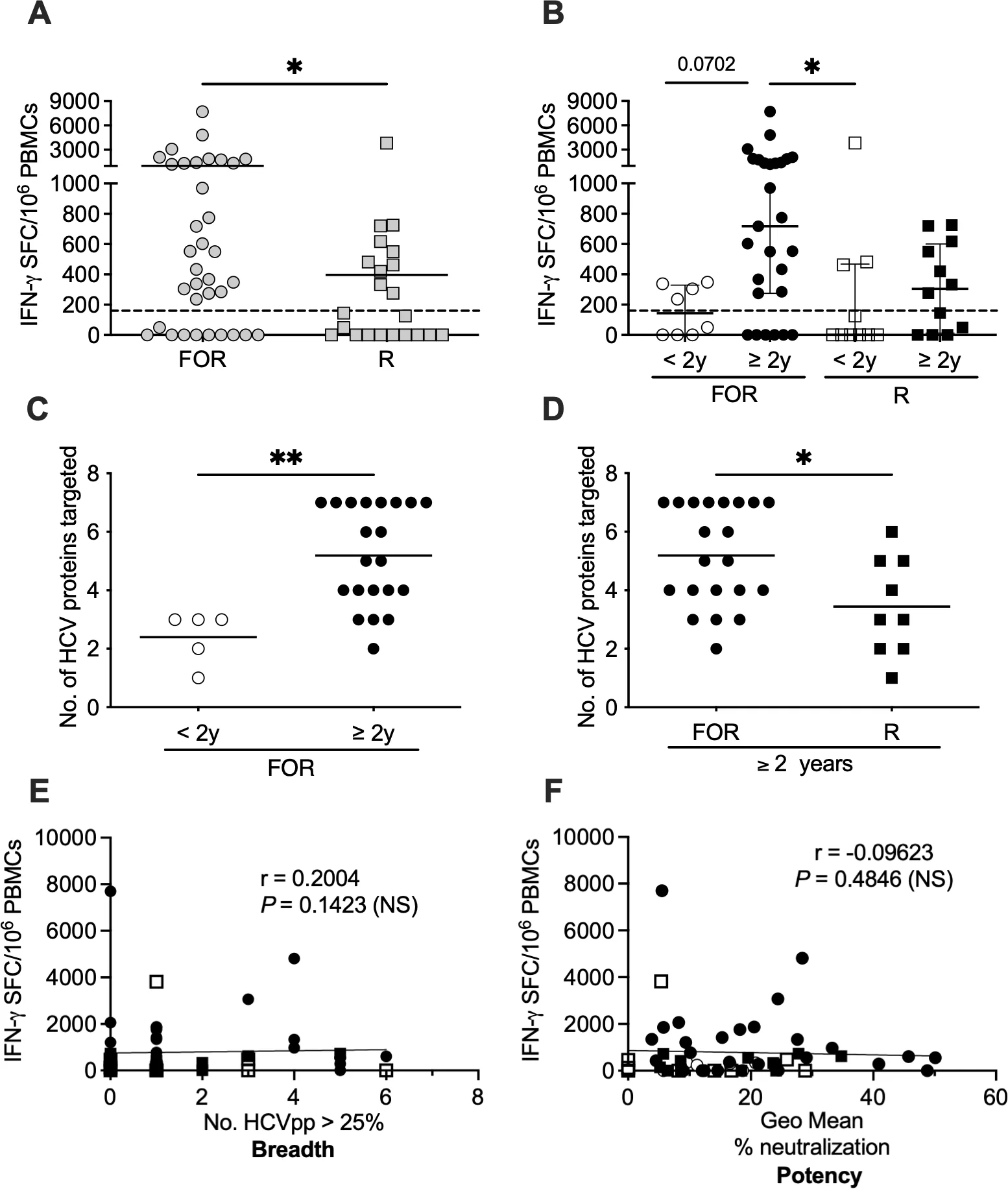
HCV-specific T cells are long-lived, broad, and present in high frequency in exposed and free of observed reinfection PWID. (**A**) Frequency of HCV-specific T cells measured by IFN-γ ELISpot in all subjects, free of observed reinfection (*n* = 34) and reinfected (*n* = 22). Two-tailed Mann-Whitney test, *P < 0.05. (**B**) Frequency of HCV-specific T cells in FOR and R subjects stratified by time since spontaneous clearance: FOR <2 years (*n* = 8), FOR ≥2 years (*n* = 26), R <2 years (*n* = 9), and R ≥2 years (*n* = 13). The dashed line indicates the minimum cut-off at 160 spot-forming cells/10^6^ PBMCs. Kruskal-Wallis test with subsequent Dunn’s multiple comparisons; *P < 0.05 (**C and D**) IFN-γ ELISpot analysis of the number of HCV proteins targeted (Core, E1/E2, p7/NS2, NS3, NS4A/B, NS5A, and NS5B) comparing FOR <2 years and FOR ≥2 years (**C**), and FOR and R subjects ≥2 years (**D**) with a positive response to HCV peptide pools (>50 SFC/10^6^ PBMCs). Two-tailed Mann-Whitney test, *P < 0.05; **P < 0.01. (**E and F**) Scatter plots for Spearman’s rank correlation between HCV-specific T cell response and neutralization breadth (**E**) or neutralization potency (**F**) for all subjects (*n* = 56); the *r*- and *P*-values are shown. NS, non-significant.

T cells are essential to control HCV infection and reinfection (9–11, 36). Therefore, we examined the HCV-specific T cell responses by evaluating IFN-γ production in an ELISpot assay upon stimulation with peptide pools spanning the entire HCV polyprotein. The frequency of the IFN-γ-producing HCV-specific T cells was significantly higher in exposed FOR compared to exposed R subjects (*P* = 0.0475) (Fig. 3A). The FOR subjects who had been exposed for ≥2 years exhibited a trend of higher magnitude of HCV-specific T cells than subjects who had been exposed for <2 years (*P* = 0.0702) (Fig. 3B). Moreover, there was a significant difference between subjects free of observed reinfection exposed ≥2 years and those reinfected exposed <2 years (*P* = 0.0488) (Fig. 3B). Notably, HCV-specific T cell responses were long-lived as they were detectable in one subject 24 years post-resolution of primary infection. Altogether, this indicates that long-term protection against reinfection is associated with a high frequency of memory HCV-specific T cells.

Next, we examined the breadth (number of HCV proteins targeted) of the IFN-γ HCV-specific T cell responses. FOR subjects who were protected from reinfection for ≥2 years exhibited significantly higher T cell breadth compared to their <2-year exposed counterparts (*P* = 0.0017) (Fig. 3C). Furthermore, our findings revealed a significant difference in the breadth of HCV-specific T cells between FOR and R subjects on long-term exposure (≥2 years) (*P* = 0.0204) (Fig. 3D). Finally, there was no correlation between the magnitude of IFN-γ-producing T cells and neutralization breadth (Fig. 3E) or potency (Fig. 3F). These results suggest that high-magnitude and broad HCV-specific T cells are associated with long-term protection from reinfection post-spontaneous resolution, and that T cells and NAbs are independent correlates of protection.

### Injection frequency correlates with neutralizing activity but not with HCV-specific T cell responses

**Fig 4:**
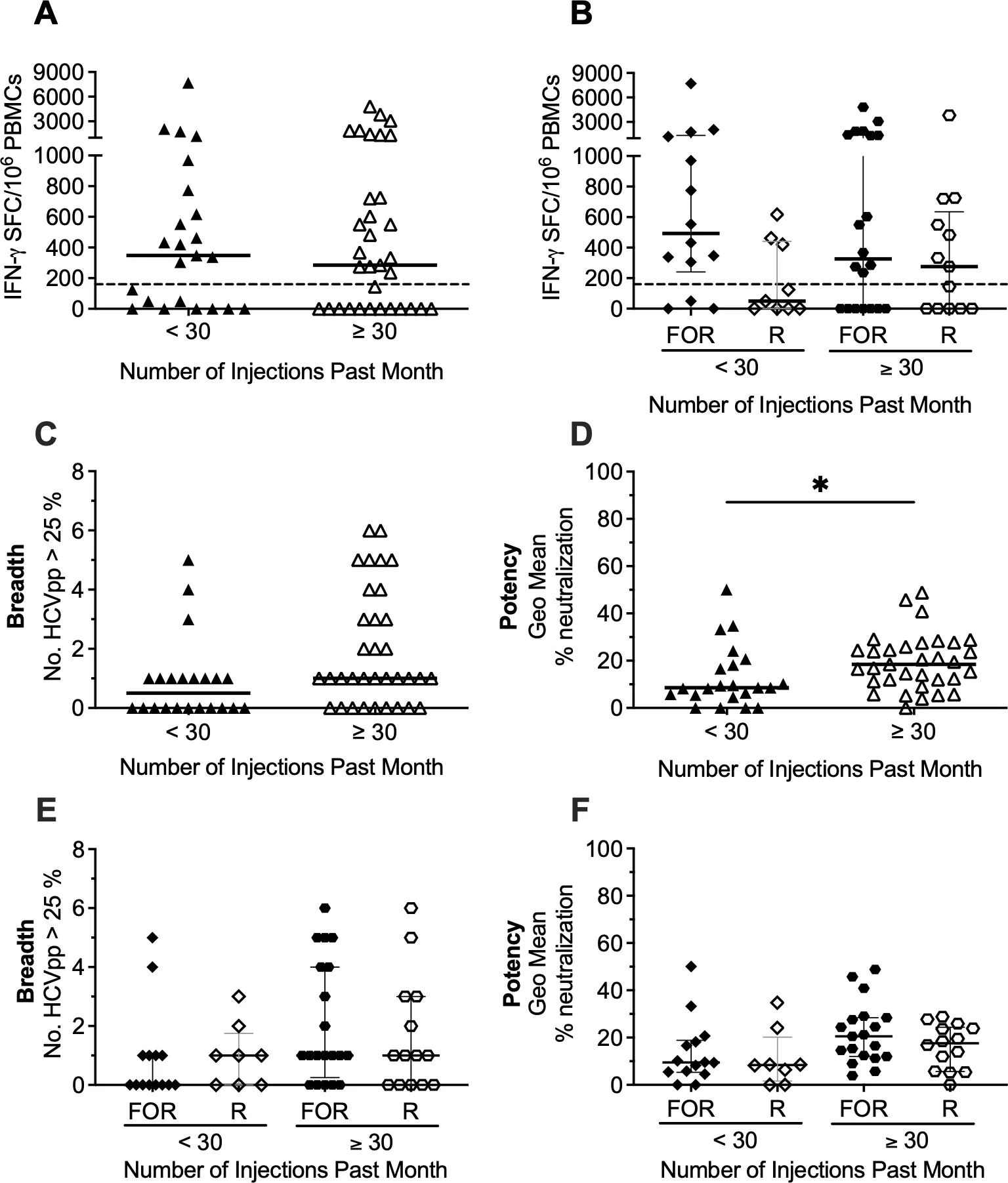
Injection frequency correlates with neutralizing activity but not with HCV-specific T cell responses. (**A and B**) Comparison of IFN-γ T cell response between participants who reported either <30 or ≥30 injections per month (**A**) and that were free of observed reinfection or reinfected and reported <30 or ≥30 injections per month (**B**). The dashed line indicates the minimum cut-off of 160 spot-forming cells/10^6^ PBMCs. (**C and D**) Comparison of neutralization breadth (**C**, number of HCVpp neutralized >25%) and neutralization potency (**D**, geometric mean of percent neutralization) between participants who reported either <30 or ≥30 injections per month. Two-tailed Mann-Whitney test. *P < 0.05. (**E and F**) Comparison of neutralization breadth (**E**) and neutralization potency (**F**) between FOR and R subjects who reported <30 or ≥30 injections in the past month.

Maintenance of high frequency of HCV-specific T cells or neutralizing activity may be due to high-risk exposures leading to short or subclinical infection(s) that boosted the immune response. We have previously demonstrated that the incidence of HCV increases with ≥30 drug injections per month (37). However, we observed a comparable percentage of FOR and R individuals with ≥30 injections (i.e., high risk) during the month preceding the time point tested (55.9% and 63.6%, respectively) (Table 1). Furthermore, there was no significant difference in the frequency of HCV-specific T cell responses detected in individuals with <30 or ≥30 injections in the past month (Fig. 4A), even when we stratified the participants into FOR and R (Fig. 4B). These results suggest no correlation between high injection frequency and T cell response and do not support the hypothesis that subclinical HCV infections could have elicited enhanced T cell responses. In contrast, neutralization breadth and potency were significantly higher in subjects reporting ≥30 injections (Fig. 4C and D). However, this was independent of their reinfection status (FOR or R) (Fig. 4E and F). Together, these results suggest that while injection frequency correlates with neutralization activity, this does not translate into protection against reinfection.

### Magnitude of IFN-γ T cell response is a significant predictor of protection in exposed PWID

**Fig 5:**
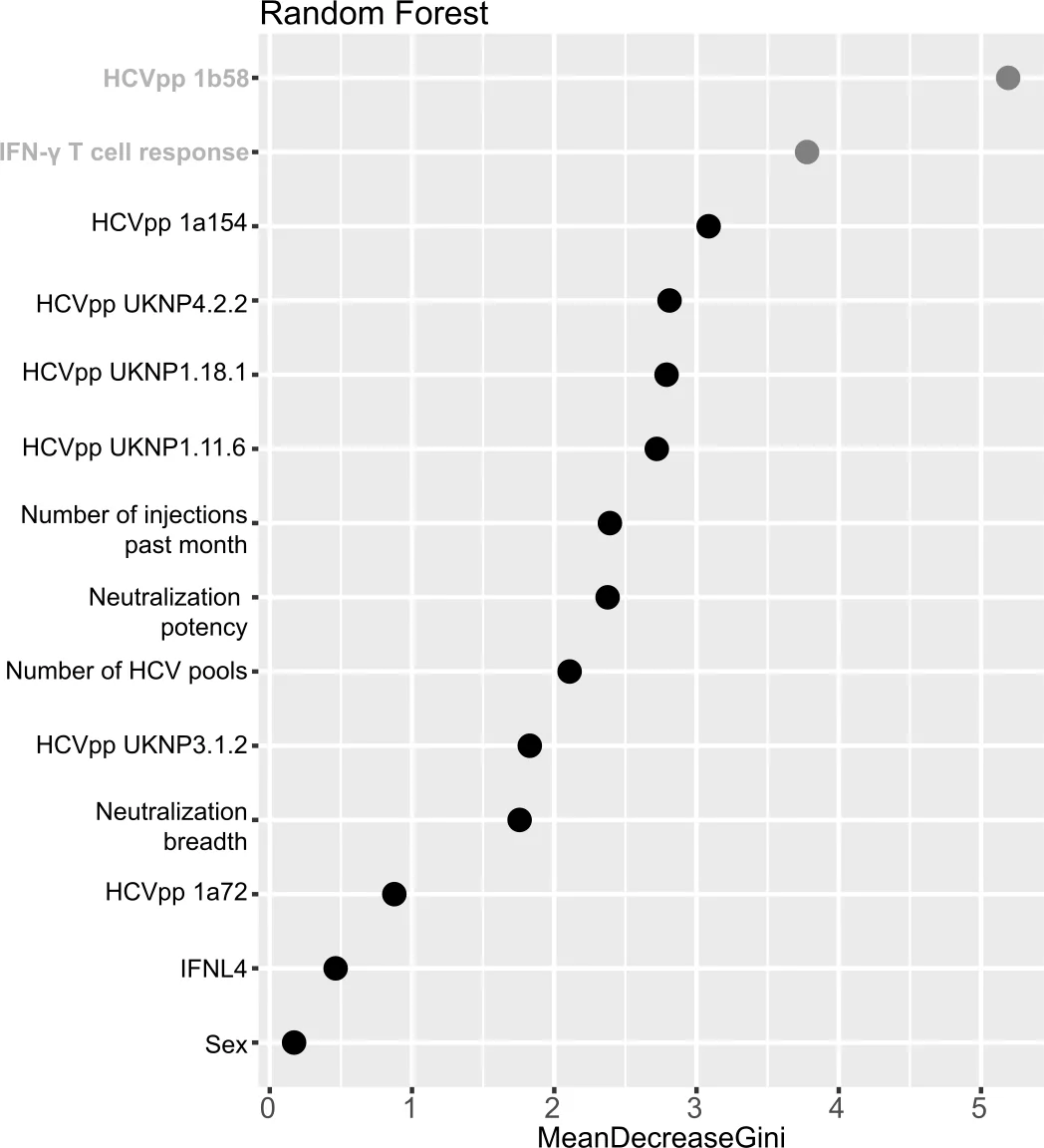
Magnitude of IFN-γ T cell response is a significant predictor of protection in exposed PWID. Multivariate random forest analysis for the prediction of status (FOR <2 years, FOR ≥2 years, R <2 years, or R ≥2 years) using relevant clinical parameters, HCVpp neutralization, and IFN-γ T cell response data as input variables. The predictors are ranked from top to bottom according to the importance of each variable. Variables with significant importance (*P* < 0.05) are shown in gray.

To determine which variables are better predictors to classify subjects into the different subgroups (FOR <2 years, FOR ≥2 years, R <2 years, or R ≥2 years), we performed a Random Forest multivariate analysis. For this analysis, we considered the neutralization of individual HCVpp, the magnitude and breadth of the T-cell response, as well as the following covariates: number of injections in the past month, *IFNL4* genotype, and sex. We also included the neutralization breadth and potency to determine if the overall NAb response could be a predictor of protection against reinfection. The goal of the Random Forest predictive model was to assess the predictive power of each of these variables in a group-agnostic fashion, rather than assessing pairwise differences of these variables within their respective groups. In addition, the Random Forest model allowed us to assess the neutralizing potential, T-cell response, as well as demographic variables together in order to distinguish the groups in question. Figure 5 shows the variables ordered by decreasing importance for the classification of subjects into the different subgroups. In this analysis, sex and *IFNL4* genotype appeared to have the lowest importance. Neutralization potency, breadth of T cell response (number of pools), and risk exposure (number of injections) appeared to be of intermediate importance, but they were not significant. Only two variables were significant (*P* < 0.05) for this prediction: neutralization of HCVpp bearing genotype 1b envelope proteins (strain 1b58, a tier 3 difficult-to-neutralize isolate), and the frequency of IFN-γ HCV-specific T cells. These results suggest that neutralization breadth and potency are not significant predictors of long-term protection from reinfection. Instead, protection could be predicted by the ability of antibodies to neutralize specific hard-to-neutralize isolates, such as 1b58, as well as by the magnitude of IFN-γ-producing HCV-specific T cell response.

### NAbs may not be protective during subsequent episodes of HCV infection in the absence of HCV-specific T cell responses

**Fig 6:**
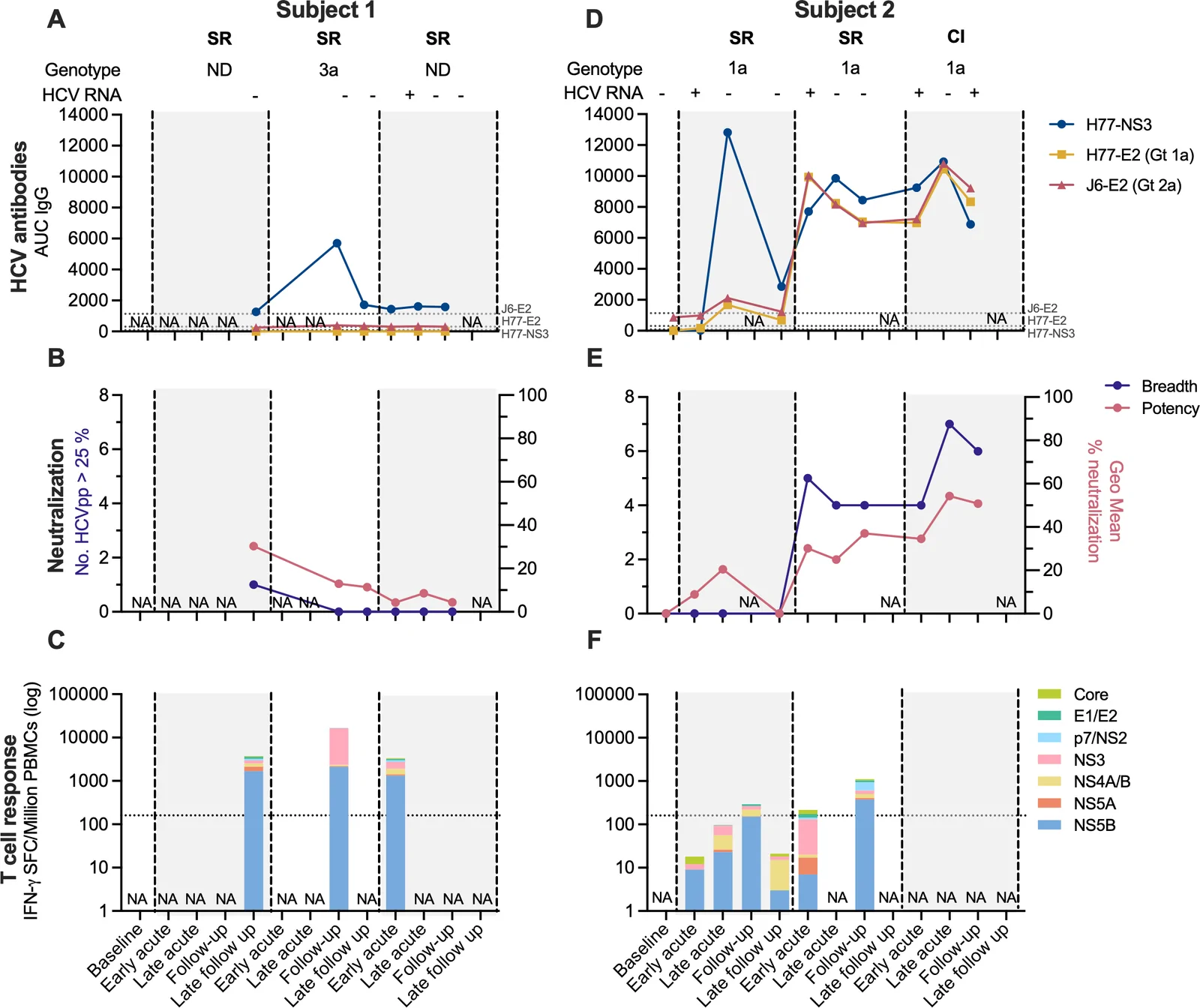
NAbs may not be protective during subsequent episodes of HCV infection in the absence of HCV-specific T cell responses. (**A and D**) Longitudinal anti-E2 (H77 and J6) and NS3 (H77) IgG responses in plasma measured by ELISA and represented as the AUC of fourfold serial plasma dilutions for Subject 1 who spontaneously resolved three infections (**A**) and Subject 2 who spontaneously resolved two infections and developed a chronic infection upon the third infection (**D**). HCV RNA and genotype are indicated on the top of the graphs. The dotted lines indicate the threshold of 84.5, 304.9, and 1,133.3 AUC for H77-NS3, H77-E2, and J6-E2, respectively. (**B and E**) Longitudinal plasma neutralization breadth (number of HCV pseudoparticles neutralized >25%, in purple) and neutralization potency (geometric mean of percent neutralization, in pink, against seven HCVpp) of Subject 1 (**B**) and Subject 2 (**E**). (**C and F**) Frequency of HCV-specific T cells (log scale) measured by IFN-γ ELISpot upon stimulation with peptide pools spanning the HCV polyprotein (Core, E1/E2, p7/NS2, NS3, NS4A/B, NS5A, and NS5B) of Subject 1 (**C**) and Subject 2 (**F**). The dotted line indicates the minimum cut-off of 160 spot-forming cells/10^6^ PBMCs. The vertical dashed lines in all panels delimit HCV infection episodes. Early acute, <3 months; late acute, 3–7 months; follow-up, 7–12 months; and late follow-up, >1 year post-estimated date of infection. SR, spontaneous resolution; CI, chronic infection; ND, not determined; and NA, sample not available.

To further analyze the contribution of T cells and NAbs to protection during multiple HCV infection episodes, we characterized the virus-specific longitudinal immune responses in two subjects who spontaneously cleared primary infection and then experienced subsequent infections (Table 2). The first subject (Subject 1) spontaneously resolved three episodes of HCV infection; the HCV genotype was only available for the second infection (genotype 3a). Plasma levels of anti-E2 (genotypes 1a and 2a) antibodies measured by ELISA were low during all the episodes of infection despite a transient increase in anti-NS3 antibodies (Fig. 6A). This was consistent with the low neutralization breadth and potency observed against the seven HCVpp tested (Fig. 6B). Notably, neutralization of HCVpp genotype 3a (UKNP3.1.2) reached its peak at only 30% during the late follow-up of the first infection (Fig. S4A). Despite subsequent reinfection with HCV genotype 3a, neutralization of this HCVpp did not increase, suggesting that viral clearance was driven by T cell immunity rather than antibody responses. In all infection episodes, we observed high frequencies of IFN-γ-producing T cells that targeted primarily the HCV non-structural proteins NS3 and NS5B (Fig. 6C). These results suggest that Subject 1 cleared all three infections primarily through T cell-mediated immunity.

**TABLE 2:** Clinical characteristics of subjects with multiple HCV infection episodes^*a*^

Characteristic	Subject 1	Subject 2	Subject 3
Sex	M	M	M
Age at HCV infection (1st/2nd/3rd)	53/57/61	25/27/28	41/42/44
HCV genotype (1st/2nd/3rd)	NA/3a/NA	1a/1a/1a	1a/1/3a
IFNλ4 rs12979860	CT	CC	CT

^*a*^ NA, not available.

The second subject (Subject 2) resolved two episodes of HCV infection but developed a chronic infection in the third one. All infection episodes were of genotype 1a. The subject exhibited high antibody titers against the E2 glycoprotein during the second and third infections (Fig. 6D). This was also reflected in the high neutralization breadth and potency against the seven HCVpp (Fig. 6E). HCVpp genotypes 1a and 4a were better neutralized (>50%) than genotype 3a and genotype 1b (Fig. S4B). Despite high neutralizing activity during the follow-up of the second episode (7 months post-EDI), persistent infection developed in the third episode. In contrast, the magnitude of T cell responses during the first and second episodes of infection was low, except at the follow-up of the second infection (Fig. 6F). These results suggest that in Subject 2, broad and potent NAbs, in the absence of a robust HCV-specific IFN-γ-producing T cell response, did not protect against the development of chronic infection upon re-exposure.

### Impaired CD8 T cell epitope recognition and late NAbs do not protect from persistent HCV reinfection

**Fig 7:**
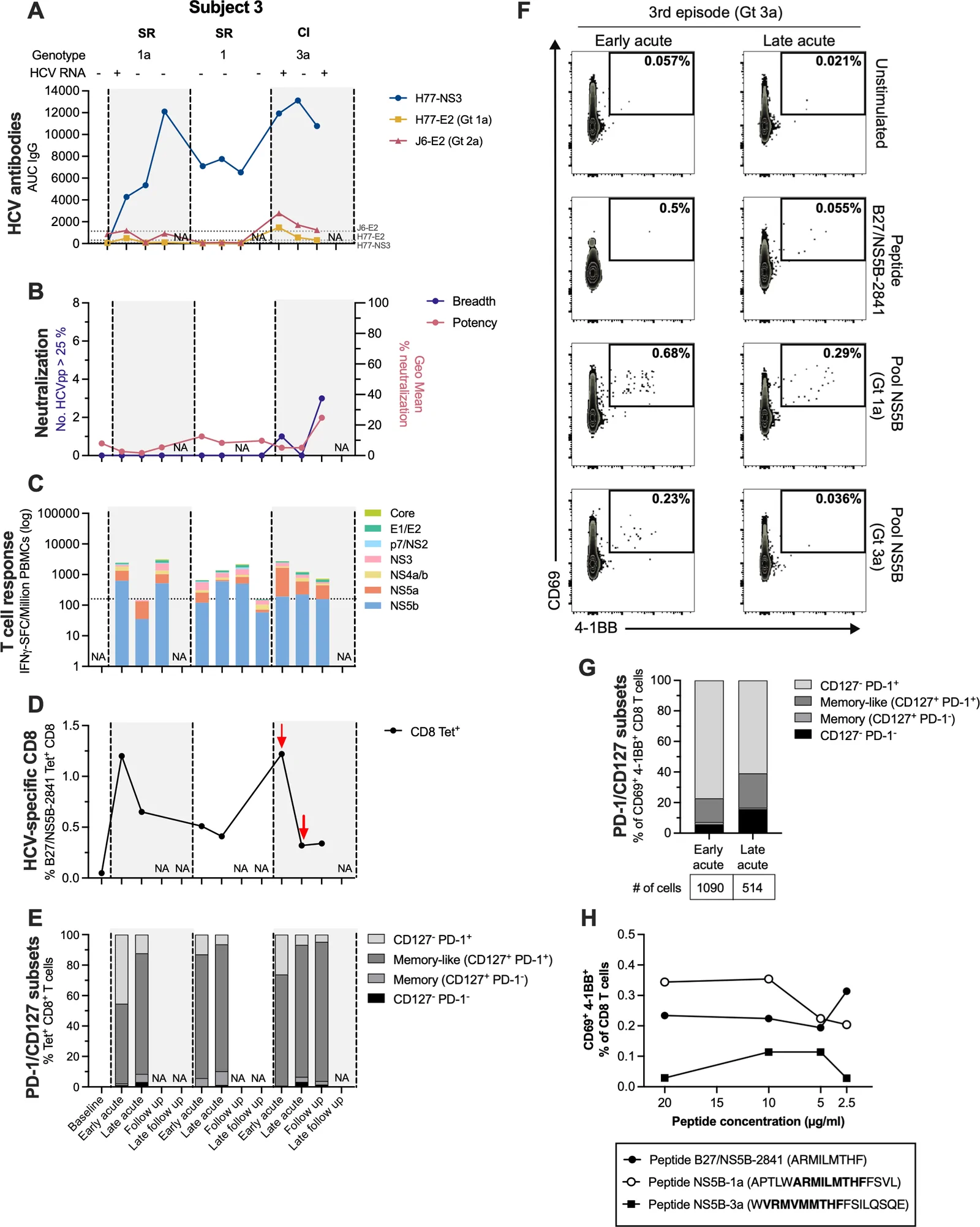
Impaired CD8 T cell epitope recognition and late NAbs do not protect from persistent HCV reinfection. (**A**) Longitudinal anti-E2 (H77 and J6) and NS3 (H77) IgG responses in plasma measured by ELISA and represented as area under the curve of fourfold serial plasma dilutions. The dotted lines indicate the threshold of 84.5, 304.9, and 1,133.3 AUC for H77-NS3, H77-E2, and J6-E2, respectively. (**B**) Longitudinal plasma neutralization breadth (number of HCVpp neutralized >25%, in purple) and neutralization potency (geometric mean of percent neutralization, in pink) against seven HCV pseudoparticles. (**C**) Frequency of HCV-specific T cells (log scale) measured by IFN-γ ELISpot by stimulation with peptide pools spanning the HCV polyprotein (Core, E1/E2, p7/NS2, NS3, NS4A/B, NS5A, and NS5B). The dotted line indicates the minimum cut-off of 160 SFC/10^6^ PBMCs. (**D**) Frequency of HCV-specific CD8 T cells identified by B27/NS5B-2841 tetramer. The red arrows indicate the time points at which the AIM assay was performed. Early acute, <3 months; late acute, 3–7 months; follow-up, 7–12 months; and late follow-up, >1 year post-estimated date of infection. (**E and G**) Bar plot showing the frequencies of PD-1/CD127 subsets as percentage of Tet^+^ (**E**) or percentage of AIM^+^ CD8 T cells from genotype (Gt) 1a pool NS5B (**G**). (**F**) Dot plot showing the AIM^+^ CD8 T cells at early and late acute phases of the third episode of infection upon stimulation with peptide NS5B_2841–2849_, Gt 1a, or 3a peptide pools NS5B. (**H**) Frequencies of AIM^+^ CD8 T cells in response to the peptide NS5B_2841–2849_ (Gt 1a) (ARMILMTHF), the peptide from the Gt 1a pool containing the epitope ARMILMTHF, and the peptide from the Gt 3a pool containing a variant sequence VRMVMMTHF. Frequencies from unstimulated cells were subtracted. The vertical dashed lines in panels **A–E** delimit HCV infection episodes. SR, spontaneous resolver; CI, chronic infection; and NA, sample not available.

Next, we studied a third subject (Subject 3) who cleared two successive infections (genotype 1a) but developed a persistent infection in the third episode (genotype 3a). Antibodies against the E2 glycoprotein (genotypes 1a and 2a) were not detected until the third episode, despite the presence of anti-NS3 at all the tested time points (Fig. 7A). This was consistent with the poor neutralization breadth and potency during the first and second episodes and a late rise in neutralizing activity during the third episode, as is characteristic of chronic HCV infection (21) (Fig. 7B). Importantly, HCVpp genotype 3a (UKNP3.1.2), the infecting genotype of the third episode, was poorly neutralized compared to genotype 1a HCVpp, suggesting that the antibody response may have been biased toward genotype 1a (Fig. S4C). This immune imprinting phenomenon was previously reported for HCV (38, 39). Conversely, the frequencies of IFN-γ-producing T cells targeting mainly NS3, NS5A, and NS5B proteins were high during all three episodes (Fig. 7C). During the third episode, Subject 3 developed a chronic infection, with the frequency of IFN-γ-producing cells peaking in the early acute phase (52 days post-EDI), followed by a progressive decline. These observations suggest that the non-sustained IFN-γ-producing HCV-specific T cell response could not protect and may have contributed to the establishment of chronic infection during the third episode.

To better characterize the HCV-specific T cell response based on the subject’s HLA background (HLA-B27^+^), we examined longitudinally the HCV-specific CD8^+^ T cells using tetramers (Tet) of the HLA-B27 immunodominant epitope NS5B-2841 (genotype 1a) (40). We observed an expansion of the Tet^+^ CD8^+^ T cells during the first and third episodes, reaching 1.2% of non-naive CD8^+^ T cells (Fig. 7D). Furthermore, we observed that the proportion of Tet^+^ CD8^+^ T cells with a memory phenotype (CD127^+^PD-1^−^) reached 8.9% in the second episode of infection, while barely 3.3% in the third one (Fig. 7E). The genotype 1a epitope (ARMILMTHF) contained in the tetramer differs by three amino acids from the infecting genotype 3a sequence (**V**RM**VM**MTHF). This suggested that the memory CD8^+^ T cells targeting this epitope may not optimally recognize the third-episode infecting sequence. Therefore, we evaluated the impact of these amino acid changes on the functionality of HCV-specific CD8^+^ T cells by performing an activation-induced marker assay, as previously described (35). We stimulated PBMCs collected during the early and late acute reinfection (52 and 109 days post-EDI during the third episode, respectively) with NS5B_2841–2849_ minimal peptide (genotype 1a), genotype 1a, or genotype 3a peptide pool NS5B (Fig. 7F). The upregulation of CD69 and 4-1BB (AIM^+^) allowed us to identify CD8^+^ T cells activated upon stimulation. The NS5B_2841–2849_ minimal peptide did not induce the activation of CD8^+^ T cells (Fig. 7F), despite the detection of these cells with the corresponding HLA-B27 tetramer (Fig. 7D). The NS5B genotype 1a pool induced the highest frequency of AIM^+^ CD8^+^ T cells at early (0.68%) and late acute (0.29%) time points. In contrast, the NS5B genotype 3a pool led to a lower (threefold) frequency of AIM^+^ CD8^+^ T cells at early acute (0.23%) and barely detectable at late acute. Upon stimulation with the NS5B genotype 1a peptide pool, more than 50% of AIM^+^ CD8^+^ T cells expressed PD-1, an inhibitory immune checkpoint marker, which may have limited full activation of the HCV-specific CD8^+^ T cell response (Fig. 7G). These results suggest that despite prior protective immune memory, impaired CD8^+^ T cell epitope recognition may have contributed to suboptimal T cell activation.

To evaluate the dose-dependent T cell activation, we stimulated PBMCs in an AIM assay with either the NS5B_2841–2849_ (ARMILMTHF) minimal peptide or the 18-mer peptides encompassing the NS5B_2841–2849_ epitope sequence from genotype 1a and 3a peptide pools (Fig. 7F). Due to the lack of samples for the early acute time point (52 days post-EDI) of the third episode of infection (Fig. 7F), we used the closest available sample (82 days post-EDI). We used decreasing peptide concentrations: 20, 10, 5, and 2.5 µg/mL (Fig. S5). We observed a dose-dependent increase in the frequencies of AIM^+^ CD8^+^ T cells with increasing concentration of the genotype 1a and 3a peptides (Fig. 7H). Moreover, the genotype 3a peptide induced the lowest frequencies of AIM^+^ CD8^+^ T cells, suggesting that amino acid changes in the genotype 3a epitope sequence may have resulted in limited recognition and subsequent CD8^+^ T cell activation. This suboptimal activation of HCV-specific memory T cells, along with the late appearance of NAbs, may have contributed to the establishment of chronic infection in Subject 3.

## DISCUSSION

We examined the contribution of T cells versus NAbs to the protection against reinfection in PWID who spontaneously resolved their primary HCV infection but maintained risk behaviors. Broad and high HCV-specific T cell responses were associated with long-term protection post-spontaneous clearance in subjects with high-risk exposure to HCV who remained free of observed reinfection. In addition, the magnitude of IFN-γ-producing T cells was a significant predictor of protection from reinfection. In contrast, while plasma neutralizing activity (breadth and potency) was high in long-term exposed subjects free of observed reinfection, only neutralization of a difficult-to-neutralize HCVpp strain (1b58) was a significant predictor of protection from reinfection. Finally, subjects who experienced subsequent HCV infection episodes could not re-clear in the absence of an effective memory T cell response despite detectable NAb activity. These results suggest that T cell immunity provides long-term protection upon re-exposure in PWID with risk behaviors.

NAbs are generally short-lived and decline following spontaneous clearance, in contrast to T cell responses that remain detectable for up to 20 years (14, 32, 36). However, in our study, NAbs positively correlated with time since spontaneous clearance. This could be partly explained by the high neutralization breadth and potency in individuals with high-risk exposure (≥30 injections). It is possible that NAbs were boosted by undetected or unproductive HCV infections that we may have missed with our 3-month follow-up testing. Despite the longer mean follow-up interval in FOR and R subjects with long-term exposure, it did not correlate with the neutralization breadth and potency (Fig. S1B and C). Long-lived HCV-specific memory B cells could have been the source of NAbs upon re-exposures, as these cells can produce antibodies several years after clearance (32, 41). It is also possible that these subjects generated a high NAb response during their primary infection that declined at a slower rate. Similar to a recent study in HIV+ high-risk men who have sex with men (42), serum neutralizing activity alone could not distinguish between free of reinfection and HCV-reinfected subjects. However, we found that neutralization of a single tier 3 (difficult-to-neutralize) HCVpp was a significant predictor of protection from reinfection. This finding suggests that antibodies targeting specific HCV isolates could be good predictors of antibody-dependent protection. Indeed, passive immunization of chimpanzees (19, 43, 44) and humanized mice (16–18, 45) with serum or monoclonal antibodies protected them against HCV challenge. Moreover, chimpanzees vaccinated with recombinant E1/E2 proteins developed protective NAbs (46, 47). Overall, antibodies can be protective, but the type of NAbs and the epitopes they target are key elements to consider in future vaccines (12, 42, 48).

Total T cell responses did not correlate with the neutralization breadth and potency post-spontaneous clearance, suggesting that HCV-specific T cells and NAbs are independent correlates of protection. In line with a previous study showing HCV-specific T cells persisted 20 years after spontaneous clearance (36), we detected long-lived HCV-specific T cell responses up to 24 years post-clearance of primary infection. Our present data also demonstrated that clearance of subsequent HCV infections is associated with a high frequency of HCV-specific T cells, but not necessarily with NAbs. Depletion studies in the chimpanzee model showed that both CD4 and CD8 T cells are essential for HCV clearance during reinfection (49, 50). In the present study, both subsets likely contributed to protection from reinfection. We show that suboptimal activation of CD8 T cells was associated with persistent infection in longitudinal samples from three subsequent HCV infections, but we could not evaluate the role of CD4 T cells, more specifically, follicular helper T cells, which correlate with the production of NAbs during primary infection and reinfection (20, 21). Finally, we and others have previously reported that T cells and NAbs can contribute to spontaneous resolution during reinfection, with distinct patterns among subjects, suggesting that both arms play a role in HCV clearance (9, 51–53). However, the extent of their contribution may vary depending on virus- and host-related factors.

We identified one individual with repeated clearance of HCV infections that became chronic despite high frequencies of IFN-γ-producing T cells. Antibodies in this subject were unable to clear the infection, possibly due to an imprinting of the humoral response by genotype 1a (38, 39). Furthermore, despite this subject being HLA-B27^+^, which is an allele associated with spontaneous clearance (40, 54), amino acid changes in the HLA-B27-restricted NS5B_2841–2849_ epitope possibly led to poor T cell activation and drove establishment of chronic infection (40, 55, 56). This suggests that the choice of antigen of a specific genotype or conserved region may be important to avoid immune escape. This argument aligns with reports from the phase I/II clinical trial of the T-cell-based vaccine that did not prevent chronic infection, potentially due to the vaccine-encoded genotype 1b antigen limiting its cross-reactivity with genotype 1a that was more prevalent in the test cohorts (22). Finally, we previously reported that healthy subjects vaccinated with that T-cell vaccine lacked a plasma cell transcriptomic signature in comparison to PWID that naturally resolved two consecutive HCV infections (51). Altogether, these findings suggest that future vaccines should include carefully designed antigens that can induce both cross-reactive T cells and NAbs.

Our study has a few limitations. First, it included samples from just 56 spontaneous resolvers, which may not have been statistically powered to identify demographic characteristics as predictors of reinfection or clearance. Additionally, 67.8% of participants were recruited as spontaneous resolvers (HCV RNA−, HCV Abs+), and therefore, we cannot exclude the possibility that some of these participants had previously cleared more than one infection. Moreover, the Montreal Hepatitis C cohort, the source of participants in our study, is ~70% male, and this is reflected in the distribution of our samples (82% male and 18% female). A previous study that collected data from nine cohorts of PWID, including ours, reported that female sex and CC (rs12979860) *IFNL4* genotype could predict re-clearance of reinfection (57), suggesting that our sample size and male-biased source are limitations. Nevertheless, despite our small sample size, the rate of spontaneous clearance of reinfection in the reinfected group was 63.6%, similar to previous reports (9, 57), and it did not vary when we further stratified participants by time since spontaneous clearance of primary infection: <2 years (66.7%) and ≥2 years (61.5%). Furthermore, we cannot exclude inaccurate disclosure of the number or frequency of injections by the participants in response to the questionnaire. Additional analyses of the viral load and infection outcomes with shorter follow-up periods and in a larger cohort may be needed.

In conclusion, we show that T cell responses and NAbs are independent contributors of long-term protective immunity against HCV to varying degrees. We propose a model where virus- and host-related factors will tilt the balance toward either a strong T cell or NAb response that will be sufficient in some situations, but most subjects will need a combination of both responses. Hence, next-generation vaccines should target both arms. Further research into the quality of NAbs and their contribution to long-term protective immunity is warranted. Given the current lack of HCV vaccines, reinfections will remain a problem among high-risk populations, including those cured with DAA, especially given that this therapy does not reconstitute fully functional T- and B-cell responses (32, 58–60). Future studies should also focus on identifying correlates of immune protection in subjects reinfected post-DAA cure.

## Data Availability

The data that support the findings of this study are available from the corresponding author, N.H.S., upon reasonable request.

## SUPPLEMENTAL MATERIAL

Supplemental material jvi.00849-26-s0001.pdf Supplemental methods, Table S1, and Fig. S1 to S5.

ASM does not own the copyrights to Supplemental Material that may be linked to, or accessed through, an article. The authors have granted ASM a non-exclusive, world-wide license to publish the Supplemental Material files. Please contact the corresponding author directly for reuse.
