# Supplementary material for: Virus-specific T cells and neutralizing antibodies are independent correlates of long-term protective immunity against hepatitis C virus
Source: J Virol. 2026 Sep 10;100(10):e00849-26. doi: 10.1128/jvi.00849-26 (PMC13712891; doi:10.1128/jvi.00849-26)
Supplement: Supplementary file 1 [file jvi.00849-26-s0001.pdf]

Supplemental Material

Virus-specific T cells and neutralizing antibodies are independent correlates of long-term protective immunity against hepatitis C virus

Elsa Gomez-Escobar[a,b,*], Asiyah Siddique[a,b,*], Omar Khedr[a], Nathalie Bédard[a], Alizée Fieffé-Bédard[a], Nourtan F. Abdeltawab[c,d], Nicol Flores[a,b], Mohamed S. Abdel-Hakeem[a,b]†, Mohamed Eisa[a], Geng Zang[a], John Lok Man Law[e]‡, Juthika Kundu[e], Michael Houghton[e], Julie Bruneau[a,f], Naglaa H. Shoukry[a,g]#

[a]Centre de Recherche du Centre Hospitalier de l'Université de Montréal (CRCHUM), Montréal, QC, Canada

[b]Département de microbiologie et immunologie, Université de Montréal, Montréal, QC, Canada

[c]Department of Microbiology and Immunology, Faculty of Pharmacy, Cairo University

[d]School of Pharmacy, Newgiza University, Giza, Egypt

[e]Li Ka Shing Applied Virology Institute, Department of Medical Microbiology and Immunology, University of Alberta, Edmonton, AB, Canada

[f]Département de médecine familiale, Université de Montréal, Montréal, QC, Canada

[g]Département de médecine, Université de Montréal, Montréal, QC, Canada

#Corresponding Author:
Dr. Naglaa Shoukry
CRCHUM
900 rue St. Denis
Montreal, QC H2X 0A9
CANADA
E-mail: naglaa.shoukry@umontreal.ca

*These authors contributed equally to this work

†Present address: Department of Pathology and Laboratory Medicine, Emory University, Atlanta, USA

‡Present address: Faculty of Medicine, Division of Biomedical Sciences, Memorial University of Newfoundland, St. John's, NL, Canada

Supplementary Methods

Random Forest analysis

The goal of this particular analysis is to identify which variables are the most significant in predicting which group (FOR > 2y, R > 2y, etc.) a particular patient belonged to. To this end, many data preprocessing steps were taken. To begin, NA (non-available) values were present in the original dataset, as is expected for such large-scale clinical-based follow-up studies. In order to overcome the challenges presented by this, KNN imputation was implemented (n = 3), using the independent variable of interest as well to aid in the prediction. KNN imputation was implemented using the VIM library in R.

Next, the optimal hyperparameters of the random forest model were achieved via grid search, using 3-fold repeated cross-validation (n = 5). In this step, optimization of hyperparameters was implemented using the libraries caret and ranger in R.

Following the generation of our final random forest model, the significance of each variable was tested using the rfPermute library in R, where the *p*-values are generated using randomly permuted values of each predictor. The model was then re-adjusted to the permuted data and compared to the original model. These random permutations were conducted 100 times in order to create a null distribution, which in turn can be used in order to determine the significance of the variable in the context of the random forest model.

References libraries in R:

1. Wickham H, Bryan J (2022). _readxl: Read Excel Files_. R package version 1.4.1, <https://CRAN.R-project.org/package=readxl>.
2. Wickham H, François R, Henry L, Müller K (2022). _dplyr: A Grammar of Data Manipulation_. R package version 1.0.10, <https://CRAN.R-project.org/package=dplyr>.

3. H. Wickham. ggplot2: Elegant Graphics for Data Analysis. Springer-Verlag New York, 2016.
4. Liaw and M. Wiener (2002). Classification and Regression by randomForest. R News 2(3), 18-22.
5. Marvin N. Wright, Andreas Ziegler (2017). ranger: A Fast Implementation of Random Forests for High Dimensional Data in C++ and R. Journal of Statistical Software, 77(1), 1-17. doi:10.18637/jss.v077.i01
6. Kuhn M (2022). _caret: Classification and Regression Training_. R package version 6.0-93, <https://CRAN.R-project.org/package=caret>.
7. Alexander Kowarik, Matthias Templ (2016). Imputation with the R Package VIM. Journal of Statistical Software, 74(7), 1-16. doi:10.18637/jss.v074.i07
8. Archer E (2022). _rfPermute: Estimate Permutation p-Values for Random Forest Importance Metrics_. R package version 2.5.1, <https://CRAN.R-project.org/package=rfPermute>.

Supplementary Tables and Figures

Supplementary Table S1. Flow cytometry antibodies

Antigen	Fluorophore	Clone	Catalog#	Supplier
CD14	V500	M5E2	561391	BD Biosciences
CD19	V500	HIB19	561121	BD Biosciences
CD3	AF700	UCHT1	557943	BD Biosciences
CD4	BV605	RPA-T4	562658	BD Biosciences
CD8	APC-H7	SK1	560179	BD Biosciences
CD279 (PD-1)	BV650	EH12	564104	BD Biosciences
CD127	BV785	A019D5	351330	Biolegend
CD69	FITC	FN50	555530	BD Biosciences
4-1BB (CD137)	PE	4B4-1	555956	BD Biosciences

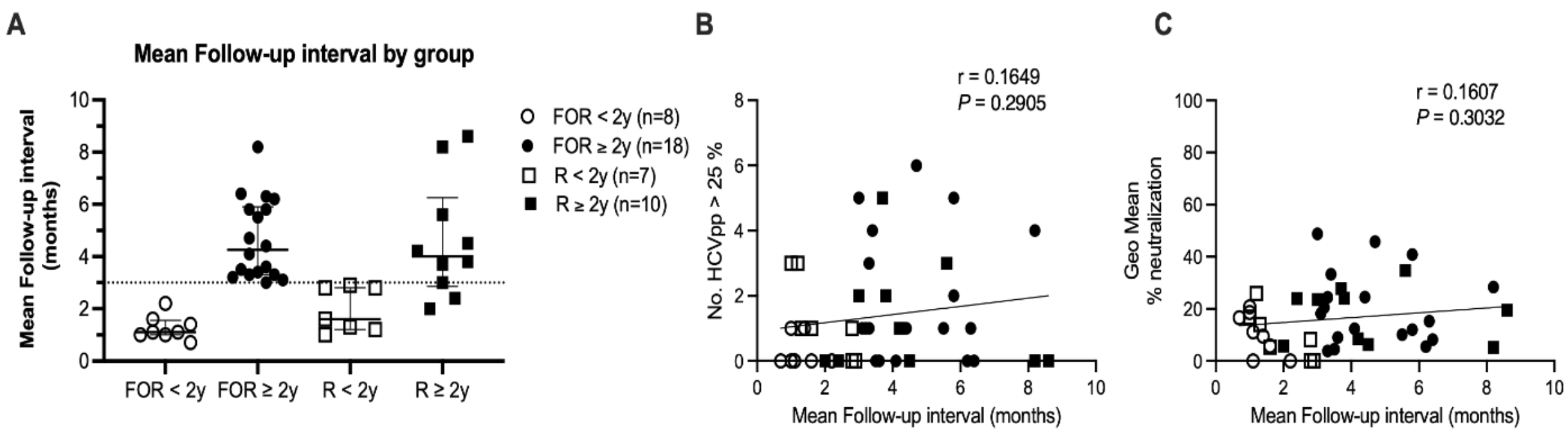

Supplementary Figure S1. Longer follow-up intervals do not broaden the NAb response. (**A**) Mean follow-up interval by group (FOR < 2y, FOR ≥ 2y, R < 2y, R ≥ 2y). Each dot represents a subject. The dotted line indicates the expected three-month follow-up. (**B** and **C**) Scatter plots for Spearman's rank correlation between mean follow-up interval and neutralization breadth (number of HCV pseudoparticles (HCVpp) neutralized > 25%) (**B**) or neutralization potency (geometric mean of % neutralization) (**C**) for all subjects (n=56), the r- and *P*-values are shown.

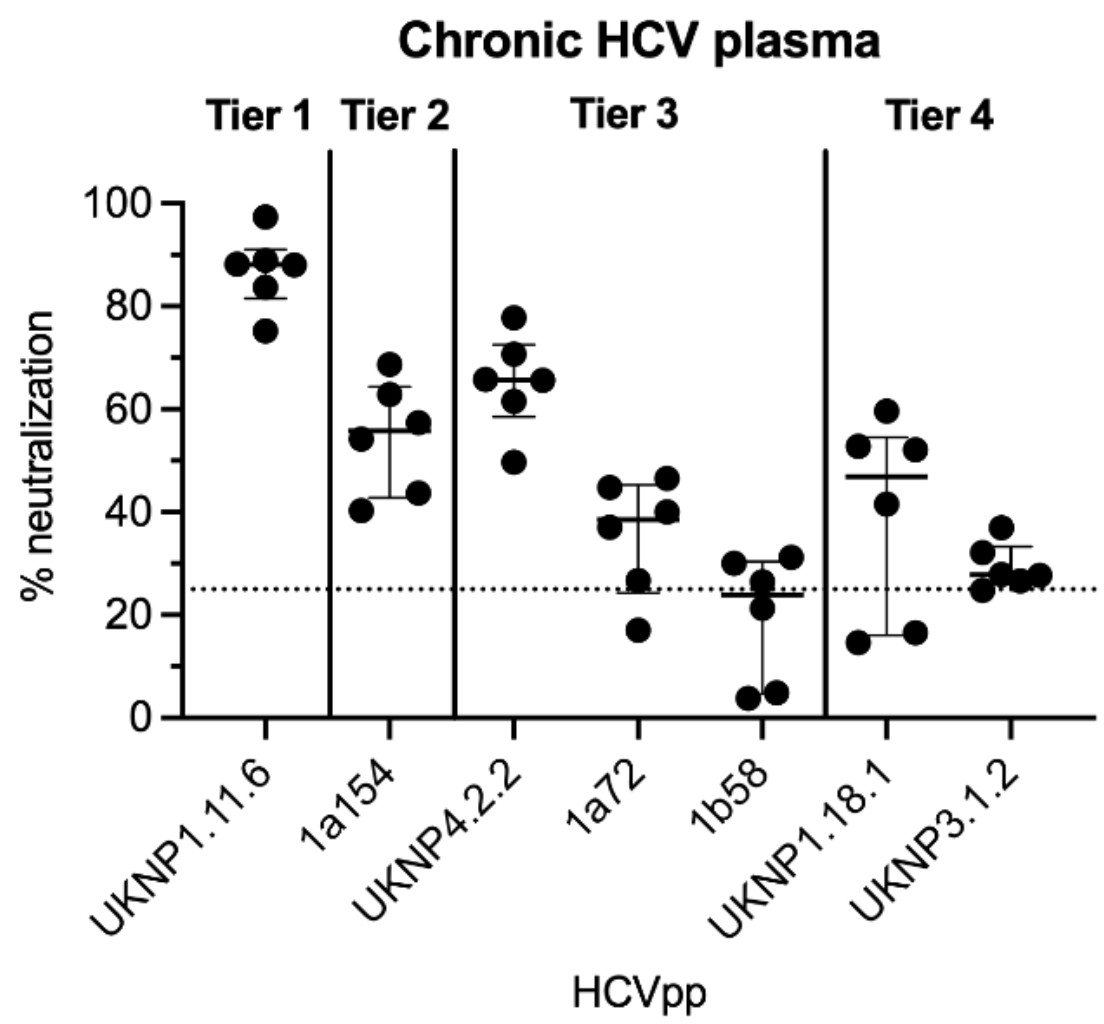

Supplementary Figure S2. Neutralization of HCVpp panel with plasma from a chronically-infected subject used as a positive control. Percentage neutralization by heat-inactivated plasma from a subject with chronic HCV infection against 7 HCV pseudoparticles from a panel. HCVpp are classified by tiers, Tier 1: UKNP1.11.6 (Gt 1a), Tier 2: 1a154 (Gt 1a), Tier 3: UKNP4.2.2 (Gt 4a), 1a72 (Gt 1a) and 1b58 (Gt 1b), and Tier 4: UKNP1.18.1 (Gt 1b) and UKNP3.1.2 (Gt 3a). For each HCVpp, dots represent independent experiments (n=6) with their correspondent medians and interquartile ranges. The dotted line indicates 25% neutralization.

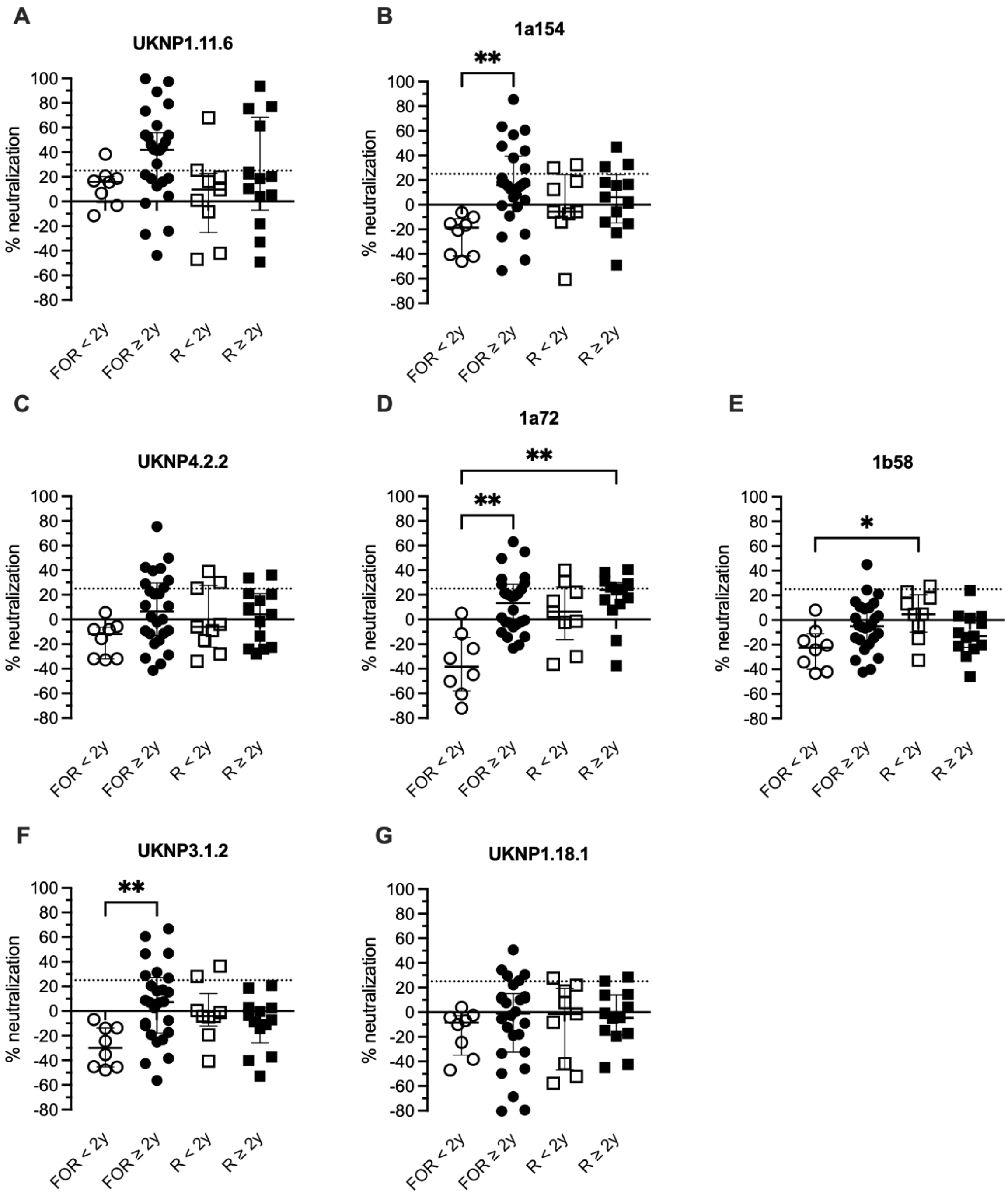

Supplementary Figure S3. Variable neutralization activity by HCV-exposed free of observed reinfection and reinfected subjects against a panel of HCVpp. Percentage neutralization by heat-inactivated plasma of free of observed reinfection (FOR) or reinfected (R) stratified by time since spontaneous clearance: FOR < 2y (n = 8), FOR ≥ 2y (n = 26), R < 2y (n = 9) and R ≥ 2y (n = 13) against 7 HCV pseudoparticles from Tier 1: UKNP1.11.6 (Gt 1a) (**A**), Tier 2: 1a154 (Gt 1a) (**B**), Tier 3: UKNP4.2.2 (Gt 4a), 1a72 (Gt 1a) and 1b58 (Gt 1b) (**C**, **D** and **E**, respectively), and Tier 4: UKNP1.18.1 (Gt 1b) and UKNP3.1.2 (Gt 3a) (**F** and **G**, respectively). The dotted lines indicate 25% neutralization. Kruskal-Wallis test with subsequent Dunn's multiple comparisons. *P < 0.05, **P < 0.01.

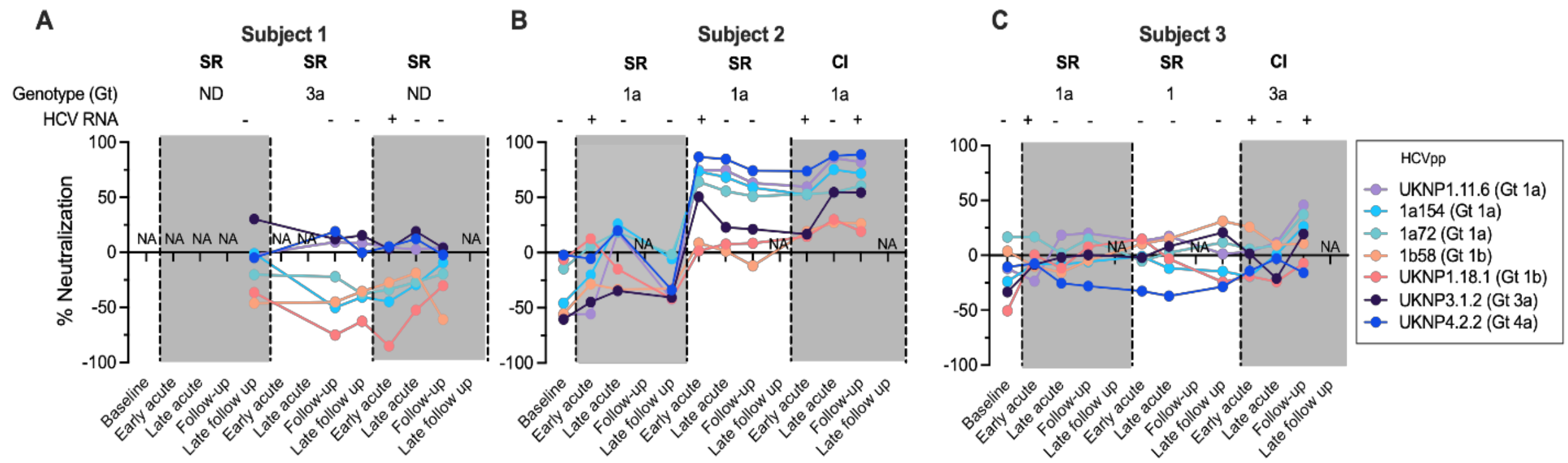

Supplementary Figure S4. Percent neutralization against HCV pseudoparticles (HCVpp) in subjects who cleared three episodes of infection. (**A-C**) Longitudinal % neutralization against seven HCVpp: UKNP1.11.6 (Gt 1a), 1a154 (Gt 1a), 1a72 (Gt 1a), 1b58 (Gt 1b), UKNP1.18.1 (Gt 1b), UKNP3.1.2 (Gt 3a), and UKNP4.2.2 (Gt 4a) for Subject 1 (**A**), Subject 2 (**B**), and Subject 3 (**C**). The infecting HCV genotype for each episode of infection and the HCV RNA (+ or -) is indicated above the graphs. The vertical dashed lines delimit HCV infection episodes. SR: spontaneous resolution; CI: chronic infection; ND: not determined; NA: sample not available; Gt: genotype.

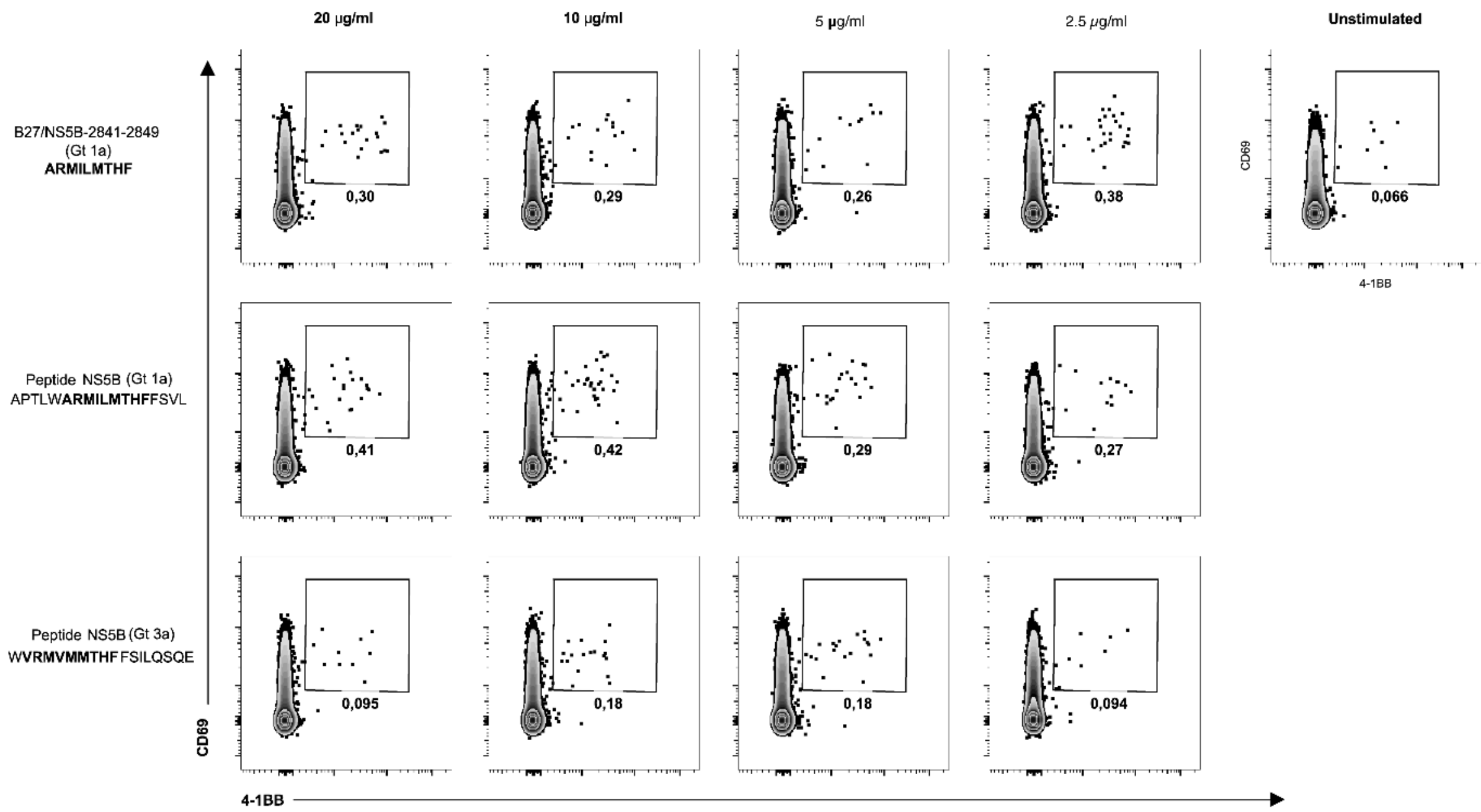

Supplementary Figure S5. Reduced recognition of variant genotype 3a epitope by CD8 T cells. Dot plots showing the frequencies of $CD69^+$ 4-$1BB^+$ (AIM^+) CD8 T cells. Each column represents a peptide concentration (20, 10, 5, and 2.5 µg/ml) and each row the peptides used to stimulate cells (References: B27/$NS5B_{2841\text{-}2849}$ ARMILMTHF and 18-mer peptide #406 NS5B genotype 1a APTLWARMILMTHFFSVL; variant: 18-mer peptide #66 NS5B genotype 3a WVRMVMMTHFFSILQSQE).
